# The Drug Treatment of Consumption

**Published:** 1894-11-03

**Authors:** 


### The Drug Treatment of Consumption.

As the season approaches when *poitrinaires* flock southwards in obedience to the prescription of physicians, who look upon climatic, dietetic, and hygienic measures as the only true treatment of consumption, those of us whose lot is cast in less pleasant places, and who cannot afford long pilgrimages in search of sunshine, are driven to think of that great mass of suffering humanity which, although afflicted with consumption, is still forced to stay at home. It is, perhaps, a misfortune for mankind that those who can speak with most authority regarding tuberculosis are largely engaged in the treatment of rich people who can afford whatever is suggested as conducive to their recovery. Fashion is responsible for many things besides the shape of bonnets, and it is unfortunately true that when leaders in medicine proclaim the all-importance of dietetic and climatic treatment a slur is cast by inference on other measures, and thus those less highly placed feel a certain hesitancy in expressing confidence in things which the *élite* esteem so little. It must be confessed, indeed, that in the sight of the pathologist drugs are fair game to poke fun at. With a honey-combed lung in his hand he may well ask what cough syrups or inhalations can do for such a condition; but we, in turn, may ask what we are to do with the unfortunates who must stay at home, who, if they can receive no help from physic, can receive no help at all.

Of all drugs for the treatment of consumption probably cough mixtures have received the greatest ridicule, attempts to cure the cough being looked upon as merely treatment of a symptom, unscientific in the highest degree. There is something to be said, however, even for cough mixtures, and that even if we accept to the full the modern teaching as to the causation of the disease. Although caused by the growth of a bacillus, nine tenths of the morbid changes in a phthisical lung are due to inflammatory processes; and are we not taught that one of the first principles in the treatment of inflammation is rest? Small step as a cough bottle may appear in the cure of a consumption, rest to the inflamed lung is at least an essential condition to its recovery. We rightly enough tell a patient to abstain from laborious work, knowing that with his feeble digestive capacity he cannot obtain nourishment to repair the resulting waste of tissue; yet cough is a harder labour than that imposed on him by any ordinary work, leaving him flushed, panting, and exhausted. We extol sleep as a sweet restorer, necessary to the maintenance of health even among the healthy, yet our poor invalid passes sleepless nights, fighting with a cough which tears him. Nor if we turn to pathology can we fail to see what an important agent is violent cough in distributing the disease throughout the lung, particles of bacillus-laden mucus being drawn back by deep inspirations along the branching bronchi, and setting up fresh foci of infection in parts hitherto untouched. These are but examples to show that the treatment of cough is not merely a treatment of symptoms, but actually goes to the root of some of the processes by which the disease is spread. Recent researches also regarding antiseptics are by no means so hopeless as some would have us believe, and it is not yet certain that drugs may not have at any rate a deterrent influence on the development of the bacilli.

Again, in regard to nutrition; if by bitter tonics we can make a man eat even two ounces more than otherwise he would, who shall say that we are not well and scientifically treating a malady which depends for its cure on the complete nutrition and the full vitality of all the tissues? Doubtless it is true that among the first and most important things in the treatment of consumption are removal of the conditions which brought it on, and the transplanting of the patient into the midst of the brightest and healthiest surroundings. Lucky are those who can have these things! But for the great mass of workers, who remain at home struggling through as best they may, oftentimes supporting themselves by their labour until near the end, other measures must be taken, and we must perforce content ourselves with what is sometimes contemptuously spoken of as mere palliative treatment. Fortunately, every year adds to our pharmacopœia new drugs and novel measures capable of giving relief to various conditions. Let us not hesitate to study and make good use of them, nor let us be led, by the undoubted and unrivalled efficacy of climatic treatment, to look on the use of other means as unworthy of our best endeavours. Scientific treatment is the application not necessarily of the best remedies, but of the best available.

